# A Cell-Based Approach to the Human Proteome Project

**DOI:** 10.1007/s13361-012-0469-9

**Published:** 2012-09-14

**Authors:** Neil L. Kelleher

**Affiliations:** 1 Department of Chemistry, Department of Molecular Biosciences, and the Chemistry of Life Processes Institute, Northwestern University, Evanston, IL 60208 USA; 2 Division of Hematology/Oncology, Robert H. Lurie Comprehensive Cancer Center, Feinberg School of Medicine, Northwestern University, Chicago, IL 60611 USA

**Keywords:** Human proteome project, Cell type, Top down proteomics, Protein isoforms, Mass spectrometry, Human genome project

## Abstract

The general scope of a project to determine the protein molecules that comprise the cells within the human body is framed. By focusing on protein primary structure as expressed in specific cell types, this concept for a cell-based version of the Human Proteome Project (CB-HPP) is crafted in a manner analogous to the Human Genome Project while recognizing that cells provide a primary context in which to define a proteome. Several activities flow from this articulation of the HPP, which enables the definition of clear milestones and deliverables. The CB-HPP highlights major gaps in our knowledge regarding cell heterogeneity and protein isoforms, and calls for development of technology that is capable of defining all human cell types and their proteomes. The main activities will involve mapping and sorting cell types combined with the application of beyond the state-of-the art in protein mass spectrometry.

## Introduction

One goal of the Human Proteome Project is to define the protein molecules that make up the human body. Such an activity could generate a reference list to better understand and detect human disease, and, with hyperfine detail, our responses to new therapeutics. What follows is a short synopsis of an idea to crystallize the Human Proteome Project (HPP) into a focused effort to map the natural structure and variation of human beings at the molecular level, much like the Human Genome Project completed a decade ago.

Whereas the decoding of the human genome involved the determination of a linear sequence of A’s, G’s, C’s, and T’s present in most of our cells, proteins are far more context-dependent. This fact, along with the complexity of highly processed protein molecules and the lack of amplification methods, forces one to define the context and scope of a compelling project that builds on the exploding knowledge of human genomes. This includes describing a clear endpoint of high value that will transform both basic and clinical research, and accelerate the delivery of societal promises made for the post-genomic practice of medicine.

## Discussion

### Current Strategies for the HPP

Launched a decade ago [1], the Human Proteome Organization, or HUPO (http://www.hupo.org/), has focused on creating knowledge bases, antibody-based reagents, and mass spectrometry-based proteomics using a “Bottom Up” analytical strategy. Using antibodies, the construction of a Human Protein Atlas (http://www.proteinatlas.org/) has yielded immunofluorescence images profiling protein expression from ~40 % of human genes. There have been two articulations of initiatives using protein mass spectrometry thus far. A “biology/disease” approach, generally linked to disease research, was first to be put forth (e.g., for the human plasma proteome, the liver, the brain, etc.) [2, 3]. This has come to be known also as a “protein-centric” or discovery approach [4]. More recently, a “gene-centric” (aka, a “chromosome-centric”, or C-HPP) approach has emerged [5, 6], with groups in many countries coordinating national efforts to map the abundance, distribution, and sub-cellular localization of proteins whose genes are co-located on the same chromosome. One additional achievement of HUPO has been to begin unifying the field of proteomics via the Proteomics Standards Initiative [7] and to provide a forum for coordinated efforts to improve cross-lab reproducibility [8]. For ease, Table 1 summarizes the current articulations of the HPP.

**Table 1:** Abbreviations Used to Notate Articulations of the Human Proteome Project (HPP)

Acronym	Project	Year proposed
B/D-HPP	Biology/disease-based Human Proteome Project^a^	2002
C-HPP	Chromosome-centric Human Proteome Project^b^	2010
CB-HPP	Cell-based Human Proteome Project	2012

^a^Also known as the organ/tissue-based HPP

^b^Also known as the gene-centric HPP)

**Figure 1:**
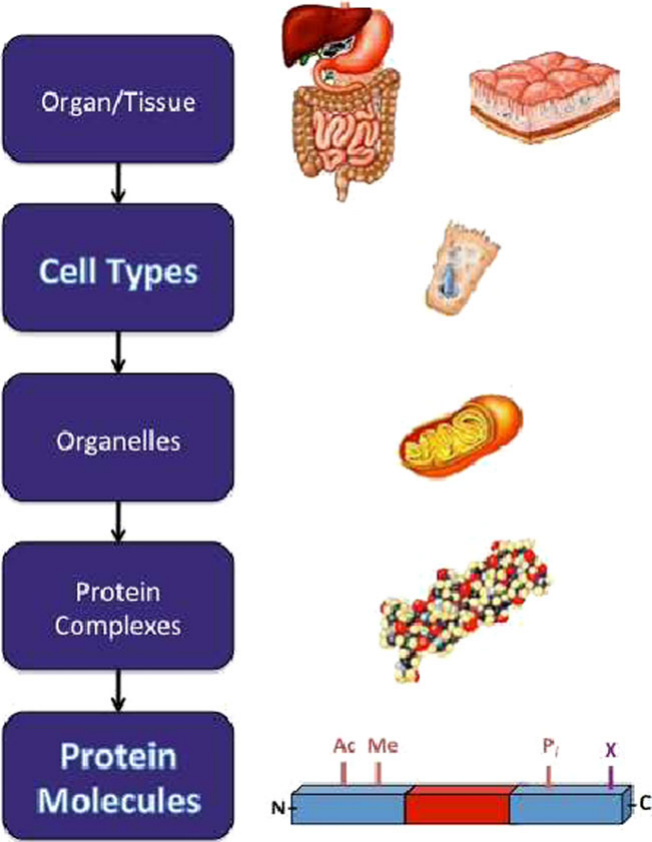
Graphical depiction of the cell-based version of the Human Proteome Project (CB-HPP)

A new context in which to place the HPP takes inspiration from a particular level of the natural organization present in the human body (Figure 1), with cell type assuming the primary, defining context for the project. With a few exceptions, it is individual cells that convert the genome into the proteome, thereby defining cell type through biomolecule expression. A cell-centric focus places a premium on knowing and classifying all the sub-types of cells in the human body. With relevance across the spectrum of human disease, a cell mapping stage naturally precedes the large-scale characterization of protein molecules (*vide infra*). This is akin to the genome mapping stages (first using genetic and then physical techniques) that dominated the first decade of the HGP (Table 2, row 2). Stimulated by the end goal and the resources to achieve it, DNA sequencing technologies underwent development at a sharply accelerated rate during this mapping stage of the project. A similar stage of technology development for quantitative measurement of protein forms is envisioned for this “Cell-Based” articulation of the HPP (or “CB-HPP”; Table 1).

**Table 2:** Comparison of the Human Genome Project (HGP) and the Cell-Based Version of the Human Proteome Project (CB-HPP)

	Aspect of Project	Human Genome Project	CB-Human Proteome Project
1	Context of cell type?	No	Yes
2	Mapping phase Required?	Yes (Genetic + Physical)	Yes (Cell-based)
3	Target size	3.2 × 10^9^ base pairs	~1 × 10^9^ protein forms
4	Leap in technology Required?	Yes	Yes
5	Model systems?	Yes (several)	Yes (e.g., microorganisms, *S. cerevisiae*)
6	Number of donors	5 or 22 people	Thousands
7	Time	15 Years	~20 Years

### Mapping Cell Types

It is clear that cellular heterogeneity is a major point of confusion in normal and disease biology, and that the textbook number of ~230 different cell types in the human body is out of date in the age of molecular medicine. The cell mapping stage of the CB-HPP can utilize a variety of cell surface markers for fluorescence assisted cell sorting (FACS) to prepare 1000 to 1,000,000 cells of high purity prior to cell-specific proteomics [9]. Further, a cell-based project calls for the large-scale discovery and validation of cell surface markers, using capture technologies for cell surface proteins [10], FACS, mass cytometry [11], RNA-seq, and other multi-parameter tools to categorize the cell types present in the human body. The Cellpedia project has generated an ontology of cell types raising the classical number of 230 to >2500 currently [12]. Given that we will add substantially to the number of cell and sample types during the cell mapping stage of the project, the number could rise to perhaps ~4000 cell types. Defining the variation of healthy cells using quantitative and isoform-resolved proteomics, both within an individual and within populations, would provide a rich basis for subsequent disease-driven research and regenerative medicine. The source of cells should be highly restricted to those isolated from primary tissue. The CB-HPP has a high bar for sampling prior to mass spectrometry-based proteomics, a will use classifiers for definition of primary cell type.

### Defining the Proteome of Specific Cell Types

**Figure 2:**
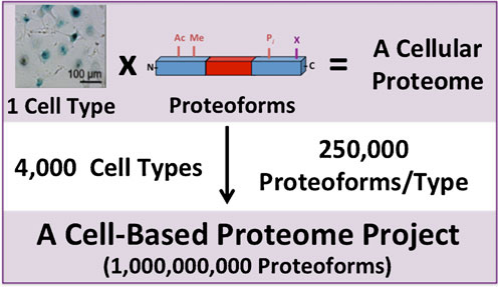
The levels of organization in the human body. The cell-based approach to the Human Proteome Project (CB-HPP) recognizes cell type as a primary context for mass spectrometry-based proteomics to measure the molecular complexity present in the body naturally. The CB-HPP also calls for accelerated development of new and emerging technologies to better define cell types and precisely catalogue whole protein molecules

This cell-based articulation of the Human Proteome Project takes inspiration, where appropriate, from the experience of the Human Genome Project (Table 2). The most analogous effort to the genome project is to provide the definitive primary structure for *Homo sapiens* at the level of protein molecules. This focused effort would involve the definition of all detectable proteoforms^1^ of carefully defined and sorted cell types from the human body. Assuming there are ~250,000 distinct proteoforms detectable in a given cell type by technologies ready within a 10-year time horizon, the whole cell-based project involves characterization of at least 1 billion proteoforms present in nondiseased cell types (Figure 2). Combined with the 10 major body fluids such as blood [13]— the core of the CB-HPP project would involve identification, characterization, and quantitation of over 1 billion detectable protein forms. The precise level of analytical depth could be adjusted once a cost versus depth model is in place prior to a production scale effort being launched around the time the C-HPP is projected to be completed in the year ~2022 [5]. To facilitate interpretation of splicing events, mutations, and coding polymorphisms, samples would be subjected to parallel genome sequencing and RNA-seq using NGS.

The Human Genome Project involved taking a grand inventory of human DNA. Similarly, the proposed CB-HPP would create definitive knowledge of cell types and the protein molecules within them. With a simplified focus on cell type and protein primary structure, the core of a focused project based on mass spectrometry can then be crafted:

- Goal: By the year 2030, to develop and apply the technology to analyze the ~1 billion primary structures of protein forms present in all the cell types and major fluids present in the human body.

This primary goal of the CB-HPP will drive development of technologies to transform the proteome from a nebulous enigma into a closed system—with knowable molecules and intelligible codes. One promising approach is the “Top Down” mass spectrometric strategy for analyzing molecules, now achievable for thousands of intact proteoforms [14]. For perspective, almost all practitioners of large-scale proteomics in discovery and targeted modes use the method of “Bottom Up” proteomics, which employs proteases to digest the primary structures of whole proteins present naturally. Clearly, both strategies can work together in a project that unifies the gene- and protein-centric articulations of the HPP. As judged by comparison with RNA-seq, Bottom Up methods are asymptotically approaching the ability to completely detect all expressed proteins (~10,000) in discovery mode from a single human cell type [15, 16]. Detection of proteoforms produced from these ~10,000 genes from carefully defined and isolated cell types then becomes the primary target for technology development in mass spectrometry-based proteomics.

This fresh and focused approach to the human proteome highlights major gaps in our current understanding of proteins and leads to a call for technological innovations (like the pioneers of genomics in the late 1980s). What combinations of coding polymorphisms, alternative splice forms, and post-translational modifications create the constellation of proteoforms present in each cell type? Once technologies are in place to answer this, we can address the question of how they vary in human disease in a deterministic and comprehensive fashion. A cell-based Human Proteome Project places a premium on defining and isolating specific cell types prior to analysis with 100 % sequence coverage for proteoforms detected at a copy number of 10 and above. Mainstream technologies in proteomics cover <20 % of the sequence space of the detectable proteome, and suffer limitations from the protein inference problem.

### An Early Example: Knowing Proteoforms of Human Histones

The human genome as presented in chromatin is 1/2 DNA and 1/2 protein by weight – and knowledge of histone forms across the ~60 million nucleosomes in diploid cells is now in view from application of the full complement of mass spectrometric methods over the years. Recently, knowledge of over a thousand distinct molecular forms of core and linker histones has been obtained by analysis of intact histones. With this bird’s eye perspective (i.e., molecular composition and approximate quantity), we have a reasonably good “basis set” of histone forms that are present down to a copy number of ~1000. While increased depth of this analysis will uncover thousands (not billions) more histone proteoforms in the future, we can already use this reference set to better understand combinations of modifications, their epigenetic contribution to diseases, and our responses to epigenetic-based therapeutics (e.g., those in development for a variety of cancers of the blood).

### Beyond Primary Structure: Capturing Protein Pleiotropism at All Levels of Organization in the Human Body

Proteins are heterogeneous and dynamic molecules in time and space. This is the reason why they are critical to our understanding of precise mechanisms in complex diseases. The dynamic nature of proteins also makes their analysis more challenging than the genome in several respects. The context of proteins in large complexes, organelles, lipid membranes, and organ/tissue type can defocus the protein analysis picture. However, recent developments do give hints of how we might proceed. For example, the canonical Top-Down experiment using mass spectrometry (i.e., complete analysis of protein primary structure) already has a next-generation counterpart, which includes characterization of the quaternary structure of megadalton protein complexes using native electrospray [17]. Also, by linking “Top Down” and “Bottom Up” flavors of mass spectrometry to separations that fractionate organelles or protein complexes, their composition can be built up using the concept of co-fractionation [18, 19]. Further, cellular and sub-cellular localization of proteins can be provided by the Human Protein Atlas, already well underway. In addition to capturing the tissue and organ context within the body (initiatives also underway in the HUPO consortium), such details on organellular localization and protein complexation form additional goals (added at considerable expense) to round out a project with the integrated resources to provide precise molecular information at each level of organization present in the human body (Figure 1).

### Comparing/Contrasting with the Human Genome Project

Without an analogue to the polymerase chain reaction (PCR) for proteins, the challenge of the human proteome requires some different strategies and tactics (Table 2). When the HGP project was begun the technological hurdles seemed insurmountable. However, the architects of the HGP recognized that when conditions are right, methodological advances come more quickly than expected. The mapping phase of the human genome provided meaningful linkages to disease research and a “Top Down” scaffold that anchored the “Bottom Up” method of whole genome shotgun sequencing. In addition to improving cell-based separations and mass spectrometric-based analyses of endogenous proteoforms, the call for disruptive technologies in proteomics would be given new voice. Assuming a cost on the same scale and growth curve of the Human Genome Project, one should demonstrate value and performance in pilot projects. While small bacteria like *H. influenzae* served nicely as models to develop whole genome shotgun sequencing, most bacterial proteins are not highly modified into a diversity of proteoforms. Despite this, such microorganisms would serve as excellent models to judge completeness and benchmark technologies capable of measuring detectable proteins with complete coverage of their primary structures (Table 2, row 5). Also, pilot projects on readily obtained human cell types can commence straight away (e.g., those of the hematopoietic system). Discovery of surface markers and methods for defining and sorting unfixed cells from solid tissue is a critical early part of the cell-mapping phase for the CB-HPP, where proteoforms are inventoried on a cell-specific basis.

### Regarding the Molecular Variation of DNA and Proteins

While the genome is quite stable and definable, there is substantial variation of it through mutation and polymorphism in populations. This variation is becoming known more fully as we reach the era of the $1000 genome [20]. The proteome has greater variation, but does not defy definition (particularly when each sample could readily have its full genome determined). Therefore, we can identify meaningful goals (vide supra), recognizing the differences between the genome and the more pleotropic proteome. Another major difference is the sampling of proteins versus DNA. For proteomics, the demands of ethical sampling would increase the requirement for a highly collaborative consortium and would extend the project in time.

### Cost and Return

The Human Genome Project involved mapping stages and much technology development stimulated by articulation and funding of the project. Even after 2003, continued stimulus through the National Institutes of Health contributed to the amazing drop of more than six orders of magnitude in cost to sequence DNA. This created over 300,000 new jobs and an estimated ~ $700 billion of economic activity [21]. A similar trajectory could be envisioned for the acquisition of information of the estimated ~1 billion proteoforms (4000 cell types × 250,000 forms/type = 1 billion protein forms). Not until the cost reaches less than $1/proteoform would a production scale effort be launched. This would provide a target to stimulate public and private sector efforts to create disruptive technologies with orders-of-magnitude increases in efficiency for discovery- and targeted-proteomics.

## Summary

The cell-based version of the Human Proteome Project, or CB-HPP, relies on two simple tenets. (1) Cells convert the blueprint of life into proteins. Therefore, a proteome project should use cell type as its primary index. (2) The “Top Down” philosophy of molecular analysis can be used in conjunction with the C-HPP project [5] to determine the complete primary structures of protein molecules on a cell-specific basis. Adherence to these tenets sharply focuses a long-term effort to create a more solid foundation for 21^st^ century biology and provides clear metrics of progress and completion.

## Outlook

This new approach to the Human Proteome Project calls for nonincremental technological leaps, and recognizes the biological hierarchy present naturally in our bodies. The CB-HPP would provide fundamental knowledge of all cells and detectable forms of protein molecules in a range of healthy human bodies. Such knowledge would revolutionize our understanding of the proteome, making it far easier and deterministic to prepare reagents and assays for diagnostics and therapeutics. For example, antibodies of the future can be constructed for targeted epitopes and even made as proteoform-specific reagents. Similarly, a drug candidate can be assessed for returning a specific constellation of proteoforms in a pathway back to a healthier state (with far better knowledge of off-target effects). Drugs and diagnostics can be devised to target specific protein molecules with a level of precision that will help drive the century of biology along the path envisioned by many decades ago. Such hyperfine control over complex biological systems was part of the original promise of the genome project; the CB-HPP can serve as a next bridge to that goal. While drawing from the HGP experience but acknowledging the strong context-dependent nature of the proteome, we may indeed see momentum gathering to develop a comprehensive understanding of just what we are at the level of protein molecules. How can we realize all our goals for the “Century of Biology” without a transformation in our molecular comprehension of the proteome?

## Question/Answer Section

Given its scope, the above proposal raises several questions and critical commentary. Upon sharing the CB-HPP concept with colleagues, common questions and issues arise, which are listed below together with responses interwoven throughout the text that follows.

**Comment/Question #1.** *The notion of cell type is flawed. Where did the estimate of 4000 cell types come from?*

**Response #1.** When asked, people discuss human cell types in a range from 230 at the low to 10^13^ at the upper end**.** There is a continuum (particularly in developmental biology and the brain), but that need not prevent us from taking a systematic approach to the classification and typing of cells. A more precise definition of cell type is akin to speciation in biology. Drawing lines and categorizing across a continuum has clear value. Such is the same required for cell types to truly know what the human body is made of. The brain is particularly diverse when thinking about cell types, something the Allen Brain Atlas is attempting to define. A recent study by Tanner and colleagues using unsupervised clustering settled on a number of 288 cell types in the human hematopoietic system [11]. Thus cell types can be defined and serve as a primary index for studies of cell-specific gene expression.

**Question #2.** *Where did the number of 250,000 proteoforms per cell type come from?*

**Response #2.** It is an estimate. There is a paradox at work here. The combinatorial nature of modifications creates a huge number of protein proteoforms. This combinatorial complexity at the whole protein level is made both simpler and more complicated when proteases are used to digest the proteome. It is simpler because of the 2^n^ combinatorial scaling of modifications on whole proteins (e.g., p53 with 15 modifications), yet more complicated with stochastic measurement of peptides and the protein inference problem. To allow precision definition of a project, the number of 250,000 is a provocative way to specify a level of depth—it is not a rigorous number and is akin to estimates of gene count in the human genome that were up to 5-fold off at early stages of the project. This number allows for the detection of the top 25 proteoforms for the products from each of the ~10,000 genes producing protein above a copy number of 25. This estimate deals with the natural complexity present by setting a metric of depth. The HGP also had population variability and a cost versus coverage problem to balance; ultimately, they chose to limit the project by both focusing on only healthy samples from a defined number of individuals. The genome is in fact quite variable in populations, but a level of depth was set (thanks in part to the Lander-Waterman Model), and the project moved into its mapping and technology development stages with estimates as a guide to frame the project and its cost.

**Question #3.** *Is there a clear endpoint for the CB-HPP?*

**Response #3.** Yes. It is what the community can define as the analytical target. While the numbers of cell types and proteoforms are estimates, they serve to conceptually define sharp edges to a Human Proteome Project.

**Issue #4.** *Judging completeness in mass spectrometry-based proteomics.*

**Response #4.** Thanks to the great advances in Bottom Up proteomics combined with RNA-seq, the full extent of gene expression in a single human cell type is coming into view, with ~9000–10,000 proteins expressed above a copy number of ~50 [15, 16]. An expectation of completeness in proteomics can now be defined. This sets the stage for ‘deep’ determination of the expressed primary structures for these gene products as whole proteoforms. Polymorphisms and mutations can be distinguished by comparison of data from next generation sequencing (NGS) of the same cell types subjected to next-generation proteomics.

**Question #5.** *Should model organisms be employed for the CB-HPP?*

**Response #5.** The benchmarking of developmental technologies is clearly needed. Using a small bacterium (such as *H. influenza*, 1.8 Mb, ~1800 ORFs) would serve as a good testing ground, with graduation to *S. cerevisiae* (~4300 expressed genes) similar to the approach taken in the Human Genome Project. However, single cell types from primary human samples can be obtained today, allowing pilot scale projects to commence in the mid-range future.

**Question #6.** *How much does it cost to measure protein forms right now using top down proteomics?*

**Response #6.** The recent study for high throughput top down proteomics cost on the order of ~ $2000 per proteoform to conduct [14]. Therefore, a >1000× decrease in cost is required before the $1/proteoform threshold could be reached. The proteome coverage, particularly for proteins >50 kDa, needs to be expanded through development of new technology at an accelerated pace.

**Question #7.** *Didn’t either the chromosome-centric or the biology/disease-driven versions of the HPP already describe this?*

**Response #7.** There have been some notions of doing cell-specific work, but a cell-based project as the primary tenant of the project has not been described widely in the disease or chromosome-centric versions of the HPP. The use of Top-Down Mass Spectrometry for interrogation of primary (or quaternary structure) has also not been described.

**Question #8.** *Shouldn’t a proteome project also target protein complexes or organelles?*

**Response #8.** Probably, but given the direct linkage between crafting a clear vision and project cost—the scope of work proposed here was crafted in a highly-focused manner, in part to counteract prior criticisms of the HPP. The measurement of intact protein complexes, or samples providing an organellular context as described in references [17–19], could be included in an expanded version of a cell-based project. Others with a clear perspective about how to sharply define such projects should put forward such proposals; this would allow funding agencies to select, which to bring into pilot stage and what the final scope of an integrated HPP might look like in the years and decades ahead.

**Question #9** ***.*** *Shouldn’t samples from various human diseases be part of all versions of the Human Proteome Project?*

**Response #9.** No. The B/D-HPP project is open-ended because disease biology is included in its articulation. Such was clearly not the case with the Human Genome Project, and the cell based-HPP follows its example closely. The Human Genome Project sequenced the genomes of five people (private effort) or 22 people (public effort), all deemed ‘healthy.’ Now that technologies for NGS of DNA/RNA are available, they are readily applied to samples from patients suffering all types of human disease (e.g., the 1000 Cancer Genomes project). However, including such samples early in a project requiring next-generation technologies for proteomics makes it very difficult to define a focused effort with precision.

**Question #10.** *What might be some intermediate milestones besides the final deliverable of the CB-HPP?*

**Response #10.** The cell types in the human hematopoetic system provide a clear focal point, not requiring the creation of single cell suspensions from human tissue. A recent study concluded that there were ~288 cell types discovered in a systematic approach using mass cytometry [11]. The final number is less important than realizing that a number can be found, even when there exists a continuum of cells undergoing differentiation from bone marrow to say, for example mature B-cells. This would serve as a proving ground for the project, with mononuclear cell types readily accessible from human blood. Determination of the proteomes from these cell types could lead to early disease-driven projects to commence in parallel with the CB-HPP focused on normal biology.

**Issue #11.** *Protein quantification in the CB-HPP.*

**Response #11.** The quantification of proteoforms is clearly part of the project. For determining a “parts list” for all types of cells, the section on histones above provides hints regarding the ability of MS to determine the relative abundance of expressed proteoforms. Absolute abundance is a separate goal, with that requiring significant technology development that would synergize nicely with the C-HPP. Quantification of proteins in a catalogue will be enabling, with the technology to accomplish this clearly of diagnostic value when analyzing samples from across the spectrum of human disease.

**Question #12.** *Is the proteome is too dynamic to draw a useful analogy to the human genome project?*

**Response #12.** No. The cellular and molecular variability of ‘normal’ at the protein level is higher than that for DNA, but this variation is not so high that it cannot be tracked in populations by introducing a type “temperature factor” for PTMs present on proteoforms (once they can be measured reasonably well). There will be protein differences that are stable and we should launch a project to define what these are at the cellular level.

**Issue #13.** *The proteome is too complex to describe a focused, transformative project.*

**Response #13.** It is true that we do not know that the Human Proteome Project (in any form) will ever become the “singularity” akin to the Human Genome Project. We seek a core focus to serve as a compelling stimulus for accelerated development of disruptive proteomics technology to improve the human condition.

**Question #14.** *How would the outcomes of the CB-HPP be used by the research community? In contrast to the genome sequence, the utility for biological experimentation of a proteome reference map is not so easily apparent.*

**Response #14.** The four main outcomes of the CB-HPP would be:

- A clear taxonomy of human cell types and their natural variation
- Technologies and reagents to define, sort, and in-situ image cell types
- Technologies for next-generation proteomics
- A reference list of proteoforms within all cell types

Like the genome project, one outcome would be enabling new technologies and knowledge bases that could be used in all areas of disease research and biology. Another expectation is that the statistical power of top down proteomics will allow stronger statistical correlations to complex phenotypes in complex populations to prevent suffering from late stage disease. Defining normal cell types and their proteoforms would then allow us define disease far more precisely, and detect it in its earliest stages more reliably and with greater return on investment. Beyond the arguments made at the end of the main text, the CB-HPP would allow us to better understand the post-translational language operative within our cells. It was partly at the level of whole proteins that evolutionary pressure has been applied, thus expanding our biology far beyond that possible with ‘only’ 20,300 genes [22]. Therefore, measuring proteoforms whole and quantitatively on a large, transformative scale has a great potential to transform 21^st^ century biology by providing a thorough “Bottom Up” knowledge of protein molecules present in healthy and diseased cells.

**Issue #15.** *The CB-HPP is a structurally based project.*

**Response #15.** So too was the genome project; population and temporal dynamics are proceeding now. The HGP was based on the primary sequence (structure) of DNA. Focusing on the primary structure of proteins allows articulation of a highly defined project. Focusing on quaternary structure as a separate articulation would add an important aspect with its associated costs. A project based on function is very difficult to frame with precision.

**Issue #16.** *It is hardly foreseeable that proteomics could be done on the scale proposed.*

**Response #16.** It was hardly foreseeable that even a single human genome could be sequenced in 1986.

## Footnotes

1 The term “*isoform*” is ambiguous, so a new term “*proteoform*” is used instead (short for “protein form” or “protein species”). Some prefer a strict use of the term “isoform” for protein products of related genes, or those arising from polymorphisms/mutations within protein-coding regions of genes. The term “*proteoform*” is used here to describe highly related protein molecules arising from all combinatorial sources of variation giving rise to products arising from a single gene. These include the genetic variations just mentioned, along with alternative splicing of RNA and post-translational modification.
